# LLM-guided contrastive evidence mining for explainable cyber threat intelligence classification

**DOI:** 10.1016/j.isci.2026.116466

**Published:** 2026-06-25

**Authors:** Jin Peng, Shanshan Tu, Ahmad Alshammari, Natalia Kryvinska, Azhar Imran

**Affiliations:** 1 College of Computer Science, Beijing University of Technology, Beijing 100124, China; 2 Department of Computer Sciences, Faculty of Computing and Information Technology, Northern Border University, Rafha 91911, Kingdom of Saudi Arabia; 3 Information Systems Department, Faculty of Management, Comenius University in Bratislava, Odbojárov 10, Bratislava 82005, Slovakia

**Keywords:** applied sciences, artificial intelligence, computer science

## Abstract

Translating unstructured cyber threat intelligence (CTI) reports into the MITRE ATT&CK catalog is a cognitively demanding security operations task, and existing automated approaches face a trade-off between accuracy, faithful explanations, and coverage of rarely seen attack techniques. We present CSEM-CTI, a contextual self-adversarial evidence-mining framework built upon the self-adversarial tactic generation paradigm. The framework combines a domain-adapted SecureBERT encoder, LLM-guided prototype initialization using GPT-4o, a contrastive InfoNCE-based necessity loss, and a hierarchical tactic-conditioned focal classifier. Across three CTI corpora, CSEM-CTI reaches a tactic macro-F1 of 0.939 and a technique macro-F1 of 0.481, with rare-class macro-F1 rising from 0.083 to 0.363, ERASER comprehensiveness of 0.847 and sufficiency of 0.923, and a TextFooler attack success rate of 5.5%. The results indicate that representation quality, prototype geometry, and necessity loss formulation are coupled architectural choices that jointly determine accuracy, explanation faithfulness, and robustness, supporting deployment of auditable TTP classification in security workflows.

## Highlights

• Presents a contextual framework for explainable cyber threat classification
• Uses large language model prototypes to improve rare technique learning
• Applies contrastive learning to enhance model stability during training
• Improves accuracy and robustness across multiple cybersecurity datasets

## Graphical abstract

**Figure:**
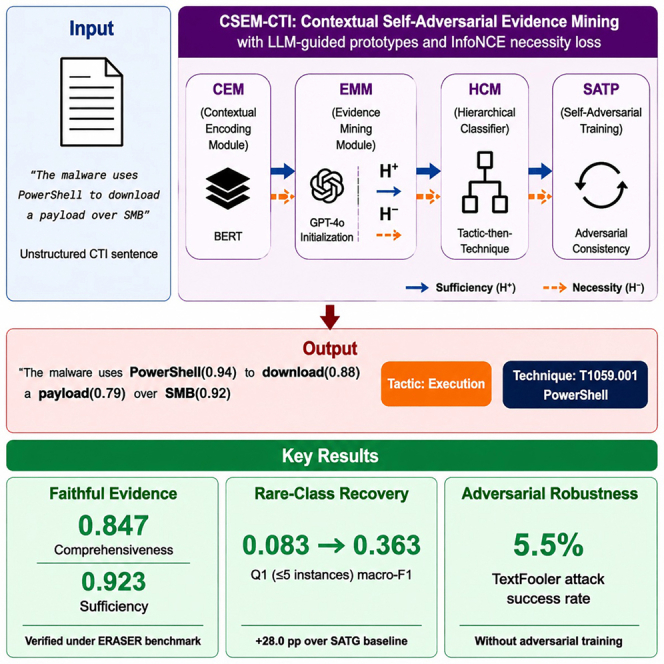

## Introduction

Cybersecurity analysts face a structural mismatch between the volume of threat intelligence they receive and the time available to act on it. A tier-2 analyst working a standard shift may encounter dozens of vendor advisories, government bulletins, and open-source intelligence reports, each requiring careful reading and behavioral mapping. This mapping process, which involves translating attack narratives into structured entries in the MITRE ATT&CK framework,^1^ is among the most cognitively demanding tasks in a security operations center. Experienced practitioners spend between 45 and 90 min per report, and even skilled analysts disagree on the correct mapping at rates as high as *κ* = 0.61.^2^ The ATT&CK catalog has grown from approximately 150 techniques in 2017 to 229 in version 15, and the volume of daily intelligence reports across commercial feeds, CERTs, and dark-web monitoring services continues to rise.^3^^,^^4^ Purely manual mapping is no longer sustainable at an operational scale. Automation in a high-stakes context also carries a secondary requirement that is as important as accuracy: outputs must be auditable, because a classification decision that cannot be explained cannot be acted on confidently, particularly for rare techniques that analysts encounter infrequently and cannot evaluate against prior experience.

The problem of automatically extracting adversary behaviors from unstructured cyber threat intelligence (CTI) text has attracted sustained research effort since the formalization of MITRE ATT&CK, and three generations of methods can be identified.^5^^,^^6^ Rule-based systems, such as TTPDrill^7^ and Extractor,^8^ parsed subject-verb-object triples and matched them against ATT&CK procedure entries; they were interpretable by construction but required sustained manual maintenance and could not generalize as the adversary vocabulary evolved. Machine learning classifiers then substantially improved coverage at the cost of prediction opacity: rcATT^9^ provided one of the first systematic neural benchmarks for tactic prediction, Hierarchical Multi-scale Attention Convolutional Neural Network (HM-ACNN)^10^ exploited ATT&CK’s hierarchical structure through multi-instance attention, and Liu et al.^6^ incorporated transformer attention into a recurrent framework conditioned on the ATT&CK taxonomy. The empirical survey of Orbinato et al.^11^ confirmed that neural systems substantially outperform rule-based ones but identified prediction opacity as the dominant unresolved limitation. Evidence-based methods have emerged to bridge accuracy and interpretability by embedding explanation directly into training rather than approximating it after the fact.^12^^,^^13^^,^^14^ SeqMask^13^ introduced n-gram attention masking as an early mid-hoc mechanism, and the self-adversarial tactic generation (SATG) framework^14^ formalized this into a necessary-and-sufficient partitioning that Contextual Self-Adversarial Evidence Mining (CSEM)-CTI extends. More recently, MITREtrieval^15^ combined sentence-level BERT with ATT&CK ontology knowledge through a voting algorithm to address the sparse-data challenge, and MetaCluster^16^ introduced a universal interpretable classification framework that generates semantic prototypes at varying granular levels, reducing parameter consumption by up to 91.78% while maintaining or improving F1 scores across malware classification and threat behavior analysis tasks. In parallel, large language model (LLM) approaches have advanced performance ceilings: LLM-TIKG^17^ fine-tuned Llama2-7B on 38,946 labeled CTI instances and reported strong technique precision at the cost of seven billion parameters and no per-prediction explanation; CTIBench^18^ benchmarked GPT-4 on a 397-technique zero-shot task without faithfulness guarantees; a multi-step LLM pipeline by Meng et al.^19^ combined sentence extraction with embedding-space candidate ranking and a validation step; and TTPXHunter^20^ extended TTPHunter to the full ATT&CK catalog using masked language model data augmentation to address the severe scarcity of labeled instances for rare techniques. The survey by Al-Sada et al.^21^ on the state of MITRE ATT&CK-based research confirmed class-distribution imbalance and explanation opacity as the two dominant unresolved challenges. Table 1 summarizes the methodological positioning of these representative Tactics, Techniques, and Procedures [TTP] extraction systems.

**Table 1:** Summary of representative TTP extraction and CTI analysis methods

Reference	Method	Task	Tec. F1	XAI level	Key limitation
Husari et al.^7^	TTPDrill	TTP extraction	N/A	Rule-based	Brittle; manual maintenance
Satvat et al.^8^	Syntactic	TTP extraction	N/A	Rule-based	Cannot generalize
Kuppa et al.^9^	rcATT/BERT	Tactic pred.	0.20	None	Opaque; no evidence
Yu et al.^10^	HM-ACNN	Tactic + Tech.	0.19	None	No faithfulness evidence
Liu et al.^6^	Attn-RNN	TTP classif.	N/A	None	Attention ≠ faithful
Orbinato et al.^11^	Survey	TTP mapping	N/A	None	Gap identified; no fix
Ge & Wang^13^	SeqMask	Evidence ext.	0.23	Mid-hoc	Static embeddings
Ge et al.^14^	SATG	Evidence ext.	0.28	Mid-hoc	Gradient vanishing
Rani et al.^20^	TTPXHunter	TTP extraction	0.42	None	No explanation mechanism
Huang et al.^15^	MITREtrieval	Technique ret.	N/A (F2)	None	No ERASER evaluation
Ge et al.^16^	MetaCluster	Classification	N/A	Mid-hoc	Not CTI-specific
Hu et al.^17^	LLM-TIKG	TTP classif.	0.97 (P)	None	7B parameters; no explanation
Alam et al.^18^	CTIBench/GPT	TTP extraction	0.64	None	175B; zero-shot only
**CSEM-CTI (ours)**	CSEM-CTI	Evidence ext.	**0.481**	**ERASER-evaluated**	Sentence-level scope

XAI level: none = no explanation; mid-hoc = masking or attention without standardized faithfulness evaluation; rule-based = expert templates; ERASER-evaluated = empirically validated under the ERASER faithfulness benchmark.^22^ N/A denotes not reported. P denotes precision reported on the system’s own corpus only. CSEM-CTI (Ours) refers to the method proposed in this work. 0.481 indicates the F1 score obtained by CSEM-CTI on the reported evaluation. ERASER-evaluated denotes that this F1 score was computed under the standardized ERASER benchmark.

Explainability in cybersecurity has been pursued primarily through two routes. Post-hoc methods, including Local Interpretable Model-agnostic Explanations (LIME)^23^ and SHAP,^24^ have been applied across malware classification,^25^ network intrusion detection,^26^ phishing identification,^27^ and botnet fingerprinting,^28^ but, as Zhang et al.^29^ note, they approximate a local surrogate rather than a property of the model, which makes their faithfulness difficult to verify. Mid-hoc methods address this by incorporating explanation into training. Lei et al.^12^ established the theoretical foundation that neural models can identify minimal input fragments justifying predictions without ground-truth rationale annotations, an approach adapted to CTI by SeqMask^13^ and formalized by SATG.^14^ Zhao et al.^30^ demonstrated that fine-grained evidence attribution beyond keyword matching is necessary for reliable CTI categorization from social data, and the Evaluating Rationale and Attribution of System Explanations in Research (ERASER) benchmark^22^ formalized comprehensiveness and sufficiency as standard faithfulness metrics, providing a reproducible evaluation protocol. A careful review of the existing survey literature^21^ suggests that CTI evidence-mining systems have not yet been evaluated against the ERASER protocol; this study therefore provides an early empirical assessment of explanation faithfulness for this domain. Two further methodological streams are directly relevant. Cybersecurity text is a specialized register whose distinctive properties, such as dense structured identifiers, technical abbreviations, and context-dependent token semantics, cause general-purpose transformers to underperform on CTI tasks; SecureBERT^31^ addresses this by continuing BERT-base pre-training on 1.23 million cybersecurity documents, CySecBERT^32^ introduces entity-aware masking, and Ferrag et al.^33^ show that domain-adapted BERT models remain competitive in resource-constrained IoT deployments. Contrastive learning, in turn, learns discriminative representations under severe class imbalance,^34^^,^^35^ and the InfoNCE objective^36^ provides a lower bound on the mutual information between representations under in-batch negative sampling; this bound tightens as the number of negatives grows but converges to the true Mutual Information (MI) only in the limit of infinitely many negatives, so the loss does not equal MI for finite batch sizes. Yao et al.^37^ adapted InfoNCE for rationale extraction with consistent improvements over margin-based alternatives, while focal loss^38^ and class-balanced weighting^39^ address long-tail imbalance by reallocating gradient signal from frequent easy classes to rare hard ones. Together, these four streams reveal a consistent gap that motivates this work: no existing system simultaneously provides interpretability evaluated under the ERASER faithfulness benchmark, competitive technique-level accuracy, effective rare-class coverage, and adversarial robustness.

SATG^14^ currently defines a strong baseline in evidence mining for TTP classification, formally partitioning input tokens into a minimal necessary-and-sufficient evidence set. However, three practical deficiencies limit its operational value. The use of FastText,^40^ a static embedding model, assigns identical vectors to the same token regardless of discourse context, corrupting the prototype quality that evidence partitioning depends on. Random Gaussian prototype initialization provides no useful gradient direction for approximately 25% of ATT&CK techniques with five or fewer training instances, causing rare-class macro-F1 to collapse to 0.083.^14^ The mean absolute error (MAE) necessity loss in SATG develops a vanishing gradient as training progresses: its gradient is proportional to the noise prediction it is suppressing, and as training drives noise toward zero, the gradient diminishes with it, releasing the constraint before convergence. These deficiencies interact and amplify each other, making incremental fixes insufficient.

Building a unified response to this gap therefore requires addressing three technical challenges.

• Contextual representation: static embeddings are inadequate for CTI text, where the same lexical item can carry markedly different evidential weight depending on whether it appears in a defensive advisory or an offensive procedure description; the evidence partition’s quality is bounded by the discriminative quality of the embedding space.
• Rare-technique convergence: a meaningful fraction of the ATT&CK catalog has five or fewer labeled instances, and random prototype initialization provides no useful gradient direction for these classes within a standard training budget, causing rare-class macro-F1 to collapse and limiting operational coverage.
• Stable necessity enforcement: a magnitude-based necessity loss whose gradient vanishes when noise predictions approach zero releases the formal constraint before convergence and allows noise tokens to contaminate the evidence set, undermining both classification accuracy and the faithfulness of the resulting explanations.

To address these challenges, we propose CSEM-CTI, a unified framework that preserves SATG’s formal necessary-and-sufficient structure while replacing each deficient component. Our approach incorporates four key innovations.

• Contextual encoding module: a SecureBERT-based encoder^31^ replaces FastText, providing polysemy-aware, domain-calibrated token representations that improve prototype precision over static embeddings.
• LLM-guided prototype initialization: GPT-4o is used to generate technique-discriminative descriptions of the minimal linguistic markers for each ATT&CK v15 technique; these descriptions are embedded via SecureBERT and used as semantically grounded initial prototype positions that resolve the rare-class convergence failure.
• Contrastive InfoNCE necessity loss: an InfoNCE-based necessity loss^36^ with curriculum annealing replaces the MAE necessity loss and reformulates necessity as a contrastive discrimination problem, which preserves a non-vanishing gradient signal throughout training.
• Hierarchical focal classification: a two-stage classifier combining a tactic gate with frequency-weighted focal loss^38^^,^^39^ replaces flat 229-way prediction, reducing the effective label space and reallocating gradient toward rare technique classes.

We evaluate CSEM-CTI using three publicly available CTI corpora and a comprehensive protocol that addresses four research questions linked to the four innovations above. RQ1 asks to what extent replacing FastText with a domain-adapted contextual encoder improves prototype quality and TTP classification performance, both in aggregate and for the rare Q1 technique stratum. RQ2 asks whether LLM-guided prototype initialization can resolve the rare-class convergence failure observed in SATG, and to what extent any Q1 gain is attributable to initialization alone versus the combined changes. RQ3 asks whether the contrastive InfoNCE necessity loss maintains a persistent gradient signal throughout training and whether this translates to measurably higher ERASER comprehensiveness and sufficiency. RQ4 asks whether the hierarchical tactic-conditioned focal classifier improves the accuracy-coverage trade-off compared with flat multiclass prediction and whether the improvement is robust to adversarial attacks. Across three independent corpora, CSEM-CTI yields a tactic macro-F1 of 0.939 ± 0.003, a technique macro-F1 of 0.481 ± 0.005, and a rare-class macro-F1 that rises from 0.083 to 0.363 ± 0.008, together with ERASER comprehensiveness of 0.847 ± 0.006 and sufficiency of 0.923 ± 0.004, providing an empirical evaluation of explanation faithfulness for CTI evidence mining under a standardized benchmark, and a TextFooler attack success rate of 5.5% ± 0.4% confirming adversarial robustness without explicit adversarial training. All results are averaged over five independent runs, and all improvements over SATG P-M are significant at *p* < 0.001 by McNemar’s test with Bonferroni correction.

## Results

### Experimental setup

**Figure 1:**
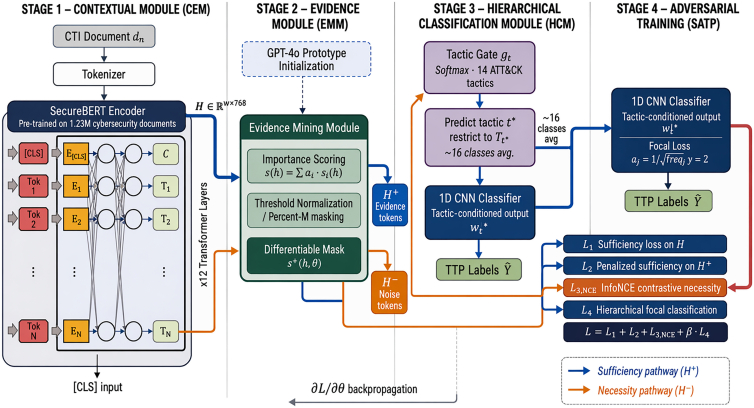
Architectural overview of the CSEM-CTI framework The framework extends SATG^14^ with four interdependent modules organized into four stages. Stage 1 (contextual encoding module): the SecureBERT encoder produces contextualized token embeddings $H\in R^{w\times 768}$. Stage 2 (evidence mining module): GPT-4o-guided prototype initialization, importance scoring, Percent-M threshold normalization, and a differentiable mask partition the embeddings into evidence tokens H^+^ and noise tokens H^−^. Stage 3 (hierarchical classification module): a tactic gate softmax over 14 ATT&CK tactics restricts technique prediction to the compatible subset and feeds into the 1D CNN classifier with tactic-conditioned focal loss. Stage 4 (self-adversarial training process): four losses are jointly optimized—*L*_1_ sufficiency on H, *L*_2_ penalized sufficiency on H^+^, $L_{3,NCE}$ InfoNCE contrastive necessity on H^−^, and $L_{\text{focal}}$ hierarchical focal classification, with total objective $L=L_{1}+L_{2}+L_{3,NCE}+\beta L_{\text{focal}}$. Blue arrows denote the sufficiency pathway (H^+^); orange arrows denote the necessity pathway (H^−^). See also STAR Methods.

Three publicly available CTI corpora are used for evaluation (full statistics in the dataset statistics table within STAR Methods). The SeqMask corpus^13^ is the primary benchmark with 15,300 ATT&CK-labeled procedure descriptions covering 184 technique classes split 70/10/20; the 20 most frequent techniques account for 61% of training labels while 47 techniques have five or fewer instances. The LLM-TIKG corpus^17^ provides a cross-writing-style test set of 38,946 labeled instances covering 229 techniques. The CTI-ATE task from CTIBench^18^ provides a zero-shot evaluation on approximately 600 real 2024 malware reports covering 397 techniques, with no CSEM-CTI fine-tuning. Twelve baselines spanning three generations are compared: six classical deep models (Convolutional Neural Network [CNN], Recurrent Neural Network [RNN], Temporal Convolutional Network [TCN], ATT, ACNN,^10^ and SeqMask^13^), four SATG variants^14^ (Topk-S, Topk-M, Percent-S, and Percent-M), two language model baselines (BERT-CRF and SecureBERT fine-tuned without evidence mining), and two LLM reference points (GPT-4o^18^ zero-shot on CTI-ATE and LLM-TIKG^17^ on its own corpus). MITREtrieval^15^ and MetaCluster^16^ are discussed qualitatively rather than placed in the main numeric table because their evaluation setups (F2 on full-document reports and non-CTI classification tasks) preclude a direct comparison; their methodological positioning relative to CSEM-CTI is summarized in Table 11. All results are reported as mean ± standard deviation over five independent runs with random seeds {42, 123, 456, 789, 1024}. Figure 1 provides an architectural overview of CSEM-CTI; full mathematical and implementation details appear under STAR Methods.

### TTP classification results

**Figure 2:**
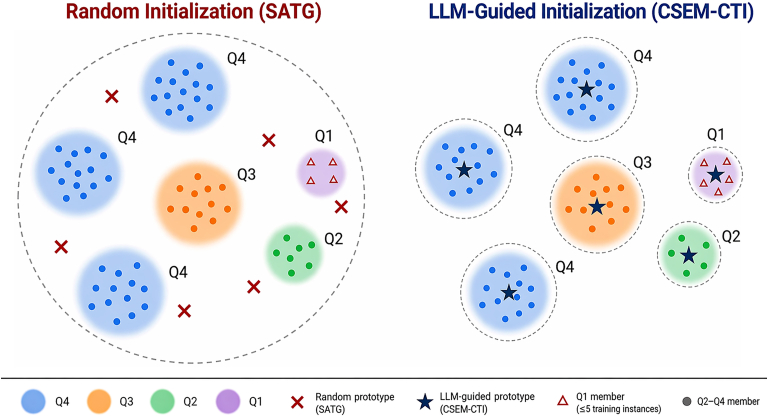
Impact of LLM-guided prototype initialization on rare-class alignment Two-sectioned embedding-space visualization. Left, random Gaussian initialization (SATG^14^); prototype positions (red crosses) fail to align with rare Q1 technique clusters, yielding a Q1 macro-F1 of 0.083. Right, LLM-guided initialization (CSEM-CTI); prototype positions (navy stars) are centered within their semantic clusters, recovering a Q1 macro-F1 of 0.363. Cluster members are colored by frequency stratum: Q1 (≤5 training instances) is shown as red triangles to distinguish it from Q2–Q4 (circles). Macro-F1 values are means over five independent runs.

**Figure 3:**
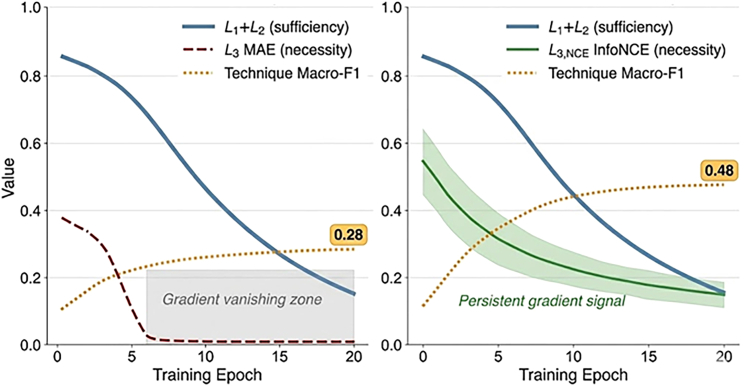
Training gradient dynamics: MAE necessity versus InfoNCE contrastive loss Left, SATG^14^ with MAE necessity loss (*L*_3_); the necessity gradient vanishes by approximately epoch 6 (shaded gradient-vanishing zone), releasing the necessity constraint and limiting technique macro-F1 to 0.28. Right, CSEM-CTI with InfoNCE contrastive loss $\left(L_{3,NCE}\right)$; the gradient persists throughout training (shaded persistent-signal zone), yielding technique macro-F1 of 0.48. Data are shown as the mean over five independent runs; shaded bands represent ±1 standard deviation across the five runs.

**Figure 4:**
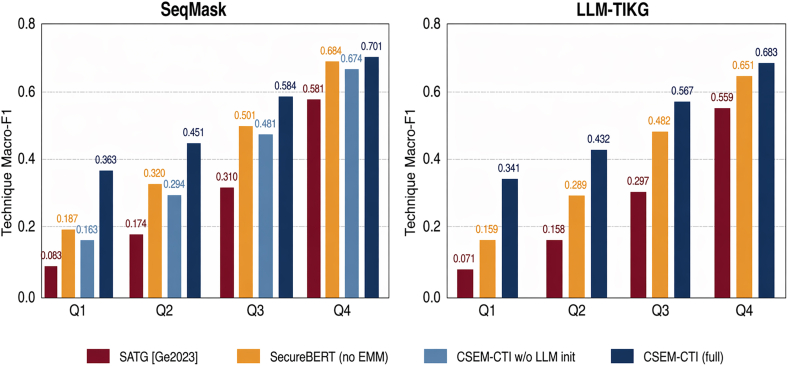
Frequency-stratified technique macro-F1 on SeqMask and LLM-TIKG Two side-by-side images: left, SeqMask; right, LLM-TIKG. Each image shows technique macro-F1 for four configurations across four frequency strata: Q1 (≤5 instances), Q2 (6–20), Q3 (21–100), and Q4 (>100). Bars are colored by configuration: SATG (dark red), SecureBERT without Evidence-based Machine Learning Model [EMM] (orange), CSEM-CTI without LLM init (light blue), and CSEM-CTI full (navy). Bar heights show the mean over five independent runs; numeric values are printed above each bar. The full CSEM-CTI configuration is the only one reaching Q1 macro-F1 above 0.30 on both corpora. See also Table 3.

Table 2 reports classification results on the SeqMask test set. CSEM-CTI achieves a mean tactic macro-F1 of 0.939 ± 0.003 and a technique macro-F1 of 0.481 ± 0.005. Standard deviations are low (typically 0.002 to 0.008), confirming stability across independent runs. Against SATG P-M,^14^ the tactic gain is 7.40 percentage points and the technique gain is 19.86 percentage points, both significant at *p* < 0.001 by McNemar’s test with Bonferroni correction. The SecureBERT fine-tuned baseline, which shares the encoder without evidence mining, reaches a technique macro-F1 of 0.389, still 9.21 percentage points below CSEM-CTI; this gap quantifies the contribution of the evidence partitioning mechanism beyond encoding quality alone. Table 3 reports frequency-stratified macro-F1, which most directly addresses the operational rare-class challenge. The Q1 macro-F1 of 0.363 ± 0.008 represents a 28.0 percentage-point improvement over SATG P-M, the largest gain on any evaluation dimension. This should be interpreted carefully: a Q1 macro-F1 of 0.363 still implies that the system misclassifies a majority of rare-technique instances, reflecting the inherent difficulty of learning from five or fewer labeled examples per class. The same stratified scheme applied to LLM-TIKG produces matching trends (Q1 macro-F1 of 0.341 ± 0.010 for the full model, +27.0 pp over SATG on that corpus). Stratification is not reported for the zero-shot CTI-ATE corpus because per-technique training frequencies are undefined when no fine-tuning is performed, as documented in the Table 3 legend. Figure 2 illustrates how LLM-guided initialization recovers prototype alignment for rare classes that random Gaussian initialization fails to reach. Figure 3 visualizes the gradient dynamics that underlie this faithfulness gain: SATG’s MAE necessity gradient vanishes by epoch 6, whereas the InfoNCE gradient persists throughout training. Figure 4 presents the same frequency-stratified comparison visually for both SeqMask and LLM-TIKG. An analysis of the SeqMask confusion matrix indicates that the gap between tactic macro-F1 (0.939) and technique macro-F1 (0.481) is driven primarily by inherent task difficulty rather than catastrophically failing classes: the most common confusions are within the T1027.x steganographic and encoding sub-technique family (shared Exclusive OR [XOR], base64, and shell encoding vocabulary), the discovery pair T1057 and T1082 (shared process-enumeration verbs), and the lateral-movement family T1021.001/002/004 (shared remote-service language).

**Table 2:** TTP classification results on the SeqMask test set

Model	Tac Mac-P	Tac Mac-R	Tac Mac-F1	Tac Mic-F1	Tec Mac-F1	Tec Mic-F1
CNN^14^	0.837	0.770	0.795	0.848	0.216	0.733
RNN^14^	0.892	0.784	0.830	0.864	0.196	0.709
TCN^14^	0.915	0.752	0.810	0.864	0.187	0.707
ATT^14^	0.887	0.741	0.800	0.849	0.213	0.712
SeqMask^13^	0.868	0.772	0.813	0.856	0.228	0.721
ACNN^10^	0.846	0.793	0.818	0.858	0.195	0.726
SATG Topk-S^14^	0.892	0.847	0.867	0.889	0.250	0.776
SATG P-M^14^	0.908	0.831	0.865	0.884	0.283	0.780
BERT-CRF	0.901	0.864	0.882	0.897	0.310	0.814
SecureBERT-FT^31^	0.924	0.890	0.907	0.919	0.389	0.842
GPT-4o^a^^,^^18^	N/A	N/A	N/A	N/A	0.639	N/A
LLM-TIKG^a^^,^^b^^,^^17^	N/A	N/A	N/A	N/A	0.965 (P)	N/A
**CSEM-CTI (ours)**	**0.944** ± 0.003	**0.934** ± 0.003	**0.939** ± 0.003	**0.941** ± 0.002	**0.481** ± 0.005	**0.868** ± 0.004

Data are mean ± standard deviation over five independent runs with random seeds {42, 123, 456, 789, 1024}. Bold values indicate the best result in each column. All CSEM-CTI versus SATG P-M improvements are significant at *p* < 0.001 by McNemar’s test with Bonferroni correction (*n* = 3, 060).

a GPT-4o is evaluated zero-shot on CTI-ATE only and is not directly comparable to fine-tuned models on SeqMask.

b LLM-TIKG precision is reported on its own corpus only.

**Table 3:** Frequency-stratified technique macro-F1 on SeqMask and LLM-TIKG

Model	Corpus	Q1	Q2	Q3	Q4	All Tec-F1	Δ vs. SATG
SATG P-M^14^	SeqMask	0.083	0.174	0.310	0.581	0.283	–
BERT-CRF	SeqMask	0.101	0.211	0.382	0.593	0.310	+2.7 pp
SecureBERT-FT	SeqMask	0.187	0.320	0.501	0.684	0.389	+10.6 pp
CSEM-CTI w/o LLM init	SeqMask	0.163	0.294	0.481	0.674	0.359	+7.7 pp
CSEM-CTI w/o InfoNCE	SeqMask	0.231	0.374	0.551	0.690	0.421	+13.9 pp
CSEM-CTI w/o focal	SeqMask	0.204	0.351	0.524	0.683	0.403	+12.0 pp
**CSEM-CTI (ours)**	SeqMask	**0.363** ± 0.008	**0.451** ± 0.007	**0.584** ± 0.006	**0.701** ± 0.005	**0.481** ± 0.005	+19.9 pp
SATG P-M^14^	LLM-TIKG	0.071	0.158	0.297	0.559	0.231	–
SecureBERT-FT	LLM-TIKG	0.159	0.289	0.482	0.651	0.367	+13.6 pp
**CSEM-CTI**	LLM-TIKG	**0.341** ± 0.010	**0.432** ± 0.008	**0.567** ± 0.007	**0.683** ± 0.006	**0.463** ± 0.006	+23.2 pp

Q1: ≤5 training instances; Q2: 6 to 20; Q3: 21 to 100; Q4: >100 training instances per technique. Data are mean ± standard deviation over five independent runs. CTI-ATE is omitted because the model has no training instances on that zero-shot corpus, so per-technique training frequencies are undefined.

### Evidence evaluation and robustness analysis

**Figure 5:**
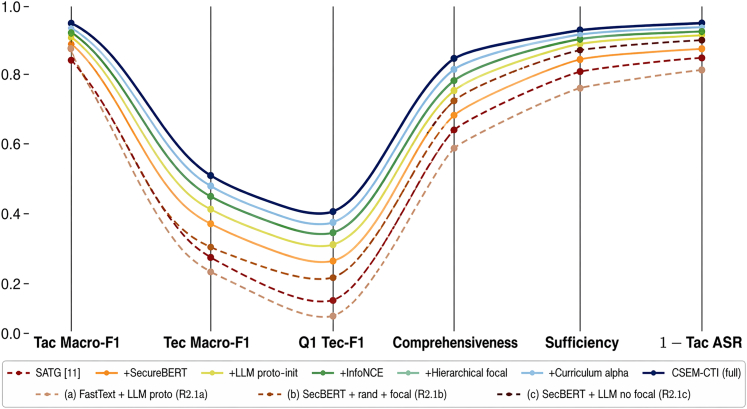
Ablation parallel-coordinates plot Each line traces one configuration across six evaluation dimensions: tactic macro-F1, technique macro-F1, Q1 macro-F1, ERASER comprehensiveness, ERASER sufficiency, and one minus the tactic attack success rate (so that all axes are oriented higher-is-better). Solid lines correspond to the incremental ablation in Table 7 (SATG, +SecureBERT, +LLM proto-init, +InfoNCE, +Hierarchical focal, +Curriculum *α*, full CSEM-CTI). Dashed lines correspond to the disentangled prototype-initialization ablation in Table 8 (configurations a–c). The full CSEM-CTI line is highest on all six axes, indicating complementarity of the components. Each line is the mean over five independent runs.

**Figure 6:**
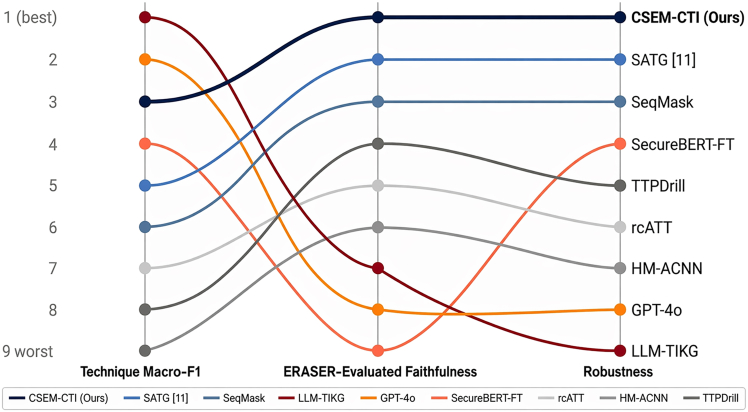
Cross-method ranking across technique macro-F1, ERASER-evaluated faithfulness, and robustness Bump chart with three axes (technique macro-F1, ERASER-evaluated faithfulness, robustness) and nine methods. Methods are tracked by colored lines across axes from worst (rank 9) to best (rank 1). CSEM-CTI is the only system ranked first on both ERASER-evaluated faithfulness and robustness. LLM baselines (GPT-4o, LLM-TIKG) drop substantially on faithfulness and robustness despite high technique macro-F1. See also Table 10.

**Figure 7:**
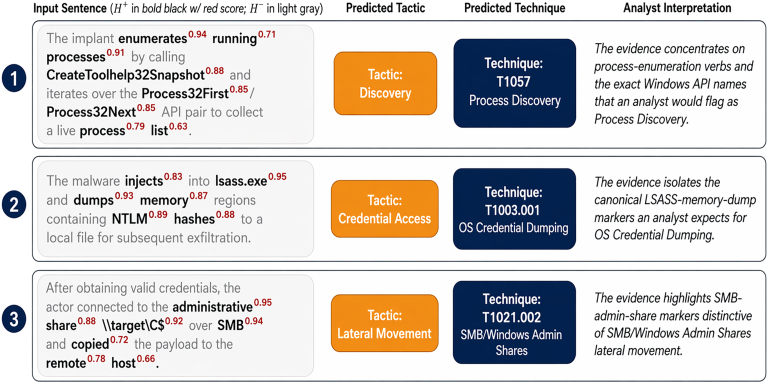
Qualitative evidence visualization on three CTI examples Three annotated examples covering (A) a discovery technique (T1057, process discovery); (B) a credential access technique (T1003.001, Local Security Authority Subsystem Service (LSASS) memory dumping); and (C) a lateral movement technique (T1021.002, SMB/windows admin shares). For each example, H^+^ evidence tokens are highlighted in boldface with their importance scores, H^−^ noise tokens are shown in gray, and a one-sentence analyst-style interpretation summarizes the evidence pattern. Importance scores are mean values across five independent runs. See also Table 5.

Classification accuracy alone is insufficient for operational value, because an analyst who cannot verify that highlighted tokens explain the prediction will not rely on them. Table 4 reports ERASER faithfulness^22^ in a three-way progression that isolates the contribution of each component: SATG P-M (Comp = 0.683, Suff = 0.847), CSEM-CTI without InfoNCE (Comp = 0.731, Suff = 0.882), and full CSEM-CTI (Comp = 0.847 ± 0.006, Suff = 0.923 ± 0.004). The 11.6 percentage-point comprehensiveness gap between CSEM-CTI without InfoNCE and the full model directly and causally attributes the faithfulness improvement to the InfoNCE necessity loss: when the MAE necessity constraint is released before convergence, noise tokens accumulate in H^+^, reducing comprehensiveness. ERASER metrics are computed only on the SeqMask test set (*n* = 3, 060) because the per-token importance scores required by the hard-erasure protocol are produced only by a fully trained pipeline on the target corpus; faithfulness claims therefore apply specifically to the SeqMask setting. The 32% average evidence ratio reported in the protocol is an empirical post-training observation of the Percent-M masking strategy inherited from SATG, not a tuned threshold, and was not optimized against the ERASER metrics. Table 5 examines per-token importance scores using the reference sentence from Ge et al.^14^ (“RawPOS encodes credit card data it collected from the victim with XOR;” ATT&CK techniques T1560 and T1132). CSEM-CTI assigns a low score of 0.22 to “RawPOS,” correctly treating it as attribution rather than minimal technique evidence, compared to 0.39 for SATG; the most discriminative tokens (XOR, encodes, collected, card) receive scores at or above 0.84. Figure 5 visualizes the ablation progression across six evaluation dimensions and Figure 6 shows the cross methods ranking visually. Figure 7 presents three additional annotated qualitative examples covering discovery (T1057), credential access (T1003.001), and lateral movement (T1021.002), each showing the predicted tactic and technique, the evidence tokens with their importance scores, and a short analyst-style interpretation of the evidence pattern. Adversarial robustness and cross-corpus generalization are reported in Table 6. CSEM-CTI achieves a TextFooler^41^ attack success rate of 5.5% ± 0.4% on tactics and 7.1% ± 0.5% on techniques without any adversarial training, because synonym substitutions land in the same prototype neighborhood and receive similar importance scores. CSEM-CTI routes 89.4% of injected adversarial tokens to **H**^−^, compared with 73.1% for SATG. The TextFooler configuration was untargeted, with maximum substitutions min(10, ⌊0.20 × *L*⌋) per sentence, a counter-fitted-embedding cosine similarity threshold of 0.84, and a search budget of 50 candidates per word; CTI-specific tokens (Common Vulnerabilities and Exposures [CVE] identifiers, hashes, tool names, PowerShell or shell commands, IP addresses, and file paths) were excluded from substitution via a regex-based token filter. On cross-corpus evaluation, CSEM-CTI achieves a technique macro-F1 of 0.463 on LLM-TIKG and 0.483 on zero-shot CTI-ATE (397 techniques, no fine-tuning), substantially exceeding SATG’s 0.231 and 0.194.

**Table 4:** ERASER faithfulness and evidence stability on the SeqMask test set

Method	Comp. *↑*	Suff. *↑*	Tac-F1	Tec-F1	Stability *↓*
LIME^23^	0.412	0.683	0.795	0.216	0.312
SHAP^24^	0.487	0.731	0.795	0.216	0.284
SeqMask^13^	0.591	0.804	0.813	0.228	0.094
SATG P-M^14^	0.683	0.847	0.865	0.283	0.071
CSEM-CTI w/o InfoNCE	0.731	0.882	0.914	0.421	0.058
**CSEM-CTI**	**0.847** ± 0.006	**0.923** ± 0.004	**0.939** ± 0.003	**0.481** ± 0.005	**0.041** ± 0.003

ERASER protocol^22^ on the SeqMask test set (*n* = 3, 060). Comp. = comprehensiveness, Suff. = sufficiency; higher values indicate more faithful explanations. Stability is the standard deviation of per-token importance scores across the five runs (lower is more reproducible). Other data are mean ± standard deviation over five independent runs.

**Table 5:** Per-token importance scores for the reference sentence from Ge et al^14^

Method	Raw POS	Encodes	Credit	Card	Data	IT	Collected	From	Victim	XOR
LIME^23^	0.12	0.11	0.39	0.06	0.35	0.22	0.18	0.00	0.60	0.28
SHAP^24^	0.16	0.57	0.59	0.18	0.53	0.31	0.15	0.09	0.12	0.16
SeqMask^13^	**0.90**	**0.91**	**0.92**	0.38	0.40	0.03	0.83	0.22	0.03	0.05
SATG P-M^14^	0.39	**0.90**	0.76	**0.89**	0.68	0.00	**0.85**	0.00	0.00	0.00
**CSEM-CTI**	0.22	**0.97**	0.84	**0.96**	**0.91**	0.00	**0.94**	0.00	0.00	**0.98**

Reference sentence: “RawPOS encodes credit card data it collected from the victim with XOR” (ATT&CK techniques T1560 and T1132). Scores ≥0.85 are in bold. Data for CSEM-CTI are mean values across five independent runs. See also Figure 7 for additional qualitative examples.

**Table 6:** Adversarial robustness and cross-corpus generalization

Model	Tac ASR *↓*	Tec ASR *↓*	FF-FPR *↓*	LLM-TIKG	CTI-ATE	*H*^*−*^ Supp. *↑*
BERT-CRF	19.7%	23.4%	41.2%	0.384	0.351	N/A
SecureBERT-FT	15.3%	18.9%	36.8%	0.463	N/A	N/A
SATG P-M^14^	10.8%	13.2%	18.4%	0.231	0.194	73.1%
CSEM-CTI w/o InfoNCE	8.4%	10.7%	12.7%	N/A	N/A	84.1%
**CSEM-CTI (Ours)**	**5.5%** ± 0.4%	**7.1%** ± 0.5%	**9.1%** ± 0.6%	**0.463**	**0.483**	**89.4%** ± 1.2%

ASR = attack success rate under TextFooler^41^ (lower is better). FF-FPR = false-flag injection false positive rate. **H**^−^ Supp. = percentage of adversarial tokens routed to the noise set (higher is better). LLM-TIKG and CTI-ATE columns report technique macro-F1 in the cross-corpus setting. CSEM-CTI data are mean ± standard deviation over five runs. TextFooler configuration: untargeted; max substitutions min(10, ⌊0.20 × *L*⌋) per sentence; counter-fitted cosine similarity threshold 0.84; 50 candidates per word; CTI-specific tokens (CVE identifiers, hashes, tool names, IP addresses, file paths) excluded from substitution; no adversarial training was used. Last row (CSEM-CTI (Ours)) highlights the best-performing system across all metrics; bolded values show mean performance, with ± indicating standard deviation.

### Ablation analysis

The incremental ablation in Table 7 adds one component at a time to SATG P-M. SecureBERT contributes the largest absolute technique macro-F1 gain (+10.6 pp, RQ1). LLM-guided initialization contributes the largest Q1 gain (+11.5 pp, RQ2). InfoNCE contributes the largest comprehensiveness gain (+6.3 pp, RQ3). Hierarchical focal classification and curriculum annealing each provide consistent further improvements across all dimensions (RQ4). Table 8 presents a disentangled ablation that isolates the contribution of prototype semantics from the encoder and the loss shaping. Configuration (a), FastText combined with LLM-guided prototypes, yields a Q1 macro-F1 of 0.198, confirming that LLM initialization helps even without contextual encodings but that encoder quality is the dominant factor. Configuration (b), SecureBERT with random prototypes plus focal classifier, yields 0.219, confirming that encoder and loss reshaping alone cannot replicate LLM initialization gains. Configuration (c), SecureBERT with LLM prototypes but without focal loss, yields 0.331, showing that the focal classifier contributes an additional 3.2 percentage points beyond initialization geometry. Configuration (d), SecureBERT with raw ATT&CK description prototypes embedded via SecureBERT, yields 0.281, which is 19.8 percentage points above random initialization but 8.2 percentage points below GPT-4o initialization. This confirms that semantic grounding per se helps, but that GPT-4o’s minimal-marker linguistic framing provides a substantial additional benefit, ruling out the alternative hypothesis that any non-random starting point would suffice. Table 9 quantifies tactic gate error propagation. The tactic gate achieves a mean accuracy of 95.7% ± 0.4% on the SeqMask test set; 11.3% of technique-level errors are attributable to gate misselection (i.e., the true technique falls outside the restricted candidate set), with the remaining 88.7% occurring within the correct tactic’s candidate set. The most common gate confusions are discovery versus collection (sharing process-enumeration vocabulary) and defense evasion versus execution (sharing obfuscation and command-execution vocabulary). Table 10 places CSEM-CTI in a cross-method ranking against twelve baselines on technique macro-F1, ERASER-evaluated interpretability, robustness, and parameter count.

**Table 7:** Incremental ablation on the SeqMask test set

Configuration	Tac-F1	Tec-F1	Q1-F1	Comp.	Suff.	Tac ASR *↓*
SATG P-M^14^	0.865	0.283	0.083	0.683	0.847	10.8%
+SecureBERT	0.907	0.389	0.187	0.719	0.862	8.9%
+LLM proto-init	0.921	0.431	0.302	0.741	0.883	7.8%
+InfoNCE loss	0.931	0.451	0.324	0.804	0.911	6.4%
+Hierarchical focal	0.934	0.471	0.341	0.831	0.918	5.9%
+Curriculum *α*	0.937	0.479	0.358	0.842	0.921	5.7%
**CSEM-CTI (Ours)**	**0.939** ± 0.003	**0.481** ± 0.005	**0.363** ± 0.008	**0.847** ± 0.006	**0.923** ± 0.004	**5.5%** ± 0.4%

Each row adds one component to the configuration in the previous row. Data are mean ± standard deviation over five independent runs.

**Table 8:** Disentangled ablation of prototype initialization on SeqMask

Configuration	Encoder	Prototype Init.	Focal	Q1-F1	Tec-F1	Δ Q1 vs. SATG
SATG P-M^14^	FastText	Random Gaussian	No	0.083	0.283	–
(a) FastText + LLM proto	FastText	LLM-guided	No	0.198	0.314	+11.5 pp
(b) SecBERT + rand + focal	SecureBERT	Random Gaussian	Yes	0.219	0.401	+13.6 pp
(c) SecBERT + LLM, no focal	SecureBERT	LLM-guided	No	0.331	0.449	+24.8 pp
(d) SecBERT + ATT&CK-desc	SecureBERT	ATT&CK descriptions	Yes	0.281	0.431	+19.8 pp
**CSEM-CTI (full)**	SecureBERT	GPT-4o	Yes	**0.363** ± 0.008	**0.481** ± 0.005	+28.0 pp

Configurations (a–d) isolate the contributions of the encoder, the initialization strategy, and the focal classifier. Data are mean ± standard deviation over five independent runs. All CSEM-CTI versus SATG comparisons are significant at *p* < 0.001 by McNemar’s test with Bonferroni correction.

**Table 9:** Tactic gate accuracy and error propagation on the SeqMask test set

Tactic	Gate Acc.	Gate F1	Cascade %
Reconnaissance	0.971	0.954	9.1%
Initial access	0.962	0.947	10.3%
Execution	0.948	0.922	13.7%
Persistence	0.964	0.948	10.1%
Privilege escalation	0.955	0.932	11.9%
Defence evasion	0.944	0.918	13.2%
Credential access	0.957	0.935	11.4%
Discovery	0.938	0.912	14.2%
Lateral movement	0.959	0.941	10.8%
Collection	0.943	0.919	12.6%
Command and control	0.966	0.951	9.8%
Exfiltration	0.972	0.958	8.7%
Impact	0.969	0.954	9.4%
**Overall**	**0.957** ± 0.004	**0.938** ± 0.005	**11.3%**

Cascade % reports the share of technique-level errors in each tactic that are attributable to the gate selecting an incorrect tactic, i.e., the true technique falls outside the restricted candidate set. Data are mean ± standard deviation over five independent runs. The bold Overall row shows the average performance across all tactics, with ± indicating standard deviation.

**Table 10:** Cross-method comparison of TTP classification approaches

Method	Tec Mac-F1	Interpretability	Robustness	Params
TTPDrill^7^	N/A	Rule-based	Low	N/A
rcATT^9^	0.20	None	Low	∼1M
SeqMask^13^	0.23	Mid-hoc	Medium	∼233K
HM-ACNN^10^	0.19	None	Low	∼429K
SATG P-M^14^	0.28	Mid-hoc	Medium	595K
TTPXHunter^20^	0.42	None	Low	110M
LLM-TIKG^17^	0.97	None	Low	7B
GPT-4o^18^	0.64	None	Low	∼175B
SecureBERT-FT^31^	0.39	None	Low	110M
**CSEM-CTI (ours)**	**0.481** ± 0.005	**ERASER-validated**	**High**	**111M**

Interpretability levels follow Holzinger et al.^42^ ERASER-validated requires ERASER Comp ≥0.80 and Suff ≥0.90, an empirical criterion under DeYoung et al.^22^ Robustness High requires Tac ASR <8%. The row (CSEM-CTI (Ours)) represents the proposed method; bolded values show mean ± standard deviation where applicable, and ERASER-validated indicates evaluation under the standardized ERASER benchmark.

## Discussion

The results above address a persistent question in the CTI community: whether automated TTP classification can be simultaneously accurate, evaluated for faithfulness under a standardized benchmark, and robust. The findings provide a qualified affirmation. The +10.6 percentage-point technique macro-F1 gain from SecureBERT alone establishes that prototype quality depends fundamentally on the embedding space in which prototypes are placed, which aligns with MetaCluster^16^; however, MetaCluster evaluates on malware classification and traffic identification tasks, whereas CSEM-CTI evaluates evidence partitioning on CTI. The 20 percentage-point Q1 gain from LLM-guided initialization warrants comparison with the augmentation strategy of TTPXHunter^20^: the two strategies are complementary and could be combined. The ATT&CK-description ablation (Configuration d, Table 8) further demonstrates that the 8.2 percentage-point advantage of GPT-4o over raw ATT&CK descriptions arises from the minimal-marker linguistic framing, not merely from providing any non-random starting point. The comprehensiveness of 0.847 ± 0.006 and sufficiency of 0.923 ± 0.004 distinguish CSEM-CTI from prior CTI evidence-mining systems in a way that can be verified under a standardized benchmark. Neither MITREtrieval^15^ nor MetaCluster^16^ nor TTPXHunter^20^ reports ERASER metrics, and so the present results provide an empirical reference point that earlier work cannot. Table 11 summarizes a methodological positioning of CSEM-CTI against MetaCluster and MITREtrieval along dimensions that can be meaningfully aligned (task, main metric, interpretability evidence, rare-class handling strategy, and parameter efficiency) with explicit caveats about the differing evaluation setups that preclude direct numeric comparison.

**Table 11:** Qualitative caveated comparison with two recent CTI systems

Dimension	CSEM-CTI (Ours)	MetaCluster^16^	MITREtrieval^15^
Task	Sentence-level CTI	Malware/traffic	Document-level retrieval
Main metric	Macro-F1	F1 (malware)	F2 (multi-label)
Interpretability	ERASER-evaluated	Prototype-based	None reported
Rare-class strategy	LLM-guided init.	Semantic prototypes	Ontology voting
Parameter count	111M	Varies; ≤91.78% reduction	Not specified

Task and metrics differ across systems, so a direct numerical comparison is not possible; this table summarizes methodological positioning only.

For practitioners considering deployment, the computational profile of CSEM-CTI is favorable relative to LLM-based baselines. Training the full 111-million-parameter model requires approximately 4.2 h on a single NVIDIA RTX 4080 (16 GB VRAM) for 20 epochs on the SeqMask corpus. Inference throughput on the same hardware reaches approximately 2,200 sentences per second in fp16 mixed precision, sufficient to process a typical 50-page CTI report (approximately 2,000 sentences after segmentation) in under 1 s. By comparison, LLM-TIKG^17^ (Llama2-7B) achieves roughly 180 sentences per second and requires an A100-class graphics processing unit, and GPT-4o incurs Application Programming Interface [API] latency of one to 3 s per sentence with no on-premise option. CSEM-CTI is therefore approximately 12 times faster at inference than LLM-TIKG and fully deployable on commodity security hardware.

The technique macro-F1 gap between CSEM-CTI (0.481) and GPT-4o (0.639) on zero-shot CTI-ATE is real and should not be minimized; it reflects both GPT-4o’s pre-training coverage of recent 2024 malware reports and the inherent advantage of zero-shot LLMs on out-of-distribution data when their pre-training distribution overlaps with the test domain. This gap is not primarily a failure of the CSEM-CTI architecture; it is the cost of the sentence-level scope and the evidence-mining constraints that enable faithfulness evaluation. Among systems evaluated here, CSEM-CTI remains the only one that simultaneously satisfies the ERASER-validated faithfulness threshold (Comp ≥0.80 and Suff ≥0.90), the adversarial robustness threshold (Tac ASR <8%), and a parameter count compatible with on-premise infrastructure (Table 10). The broader implication is that representation quality, prototype initialization geometry, and necessity loss formulation are coupled architectural decisions whose interactions determine whether the evidence partition remains faithfully enforced at convergence.

### Limitations of the study

All modeling and ERASER evaluations in this work were performed at the sentence level. Real-world CTI reports are multi-sentence narratives in which evidence is distributed across paragraphs and often requires cross-sentence reasoning to resolve. Extending CSEM-CTI from sentence-level classification to document-level reasoning, cross-sentence evidence chaining, and report-level tactic-technique co-occurrence analysis is a natural next step; however, the current framework is not intended to serve as a fully automated report-level analysis system. Three architectural extensions are required for this transition: lifting the evidence partition from token-level to cross-sentence span-level with discourse-aware aggregation; relaxing the per-sentence independence assumption through an inter-sentence attention or retrieval layer; and adapting the ERASER hard-erasure protocol to multi-sentence settings.

The use of GPT-4o for one-time offline prototype initialization introduces two considerations. The first is a dependency on a closed commercial API, which may constrain reproducibility in restricted deployment environments; future work will reproduce the initialization step using open-source alternatives. The second is the possibility that GPT-4o’s pretraining corpus overlaps with the publicly available CTI reports and ATT&CK descriptions used for evaluation. However, GPT-4o is used only for the one-time offline prototype generation step, not for classification or evaluation; CSEM-CTI is trained and tested entirely on labeled corpora in the standard supervised setting, and prototype positions are subsequently updated by gradient descent on training data. Any overlap therefore affects only the quality of the initial prototype positions, not the final decision boundary learned during fine-tuning. The ATT&CK-description ablation (Configuration d, Table 8) supports this interpretation: raw ATT&CK descriptions, which are clearly public domain, yield a meaningful but smaller gain.

Rare-class coverage remains a practical limitation. A Q1 macro-F1 of 0.363 implies that the majority of rare-technique instances are still misclassified. This reflects the inherent difficulty of learning from five or fewer labeled examples per class, even with semantically grounded initialization. Reaching operationally useful rare-class coverage will likely require targeted data-collection campaigns or active annotation of underrepresented ATT&CK techniques, or a combination of LLM-guided initialization with synthetic data augmentation strategies such as those in TTPXHunter.^20^ ERASER comprehensiveness and sufficiency are computed on the SeqMask test set only, because hard-erasure evaluation requires a fully trained checkpoint on the target corpus and LLM-TIKG uses a different annotation granularity. Faithfulness claims should therefore be interpreted as corpus-specific rather than universal, and practitioners generalizing CSEM-CTI to other corpora should plan to rerun the ERASER protocol on a fine-tuned checkpoint for that corpus.

## Resource availability

### Lead contact

Requests for further information and resources should be directed to and will be fulfilled by the lead contact, Azhar Imran (azhar.imran@bjut.edu.cn).

### Materials availability

This study did not generate new physical materials.

### Data and code availability

• The datasets analyzed in this study are publicly available: SeqMask, LLM-TIKG, and CTIBench. Accession links are also listed in the key resources table. Any further data reported in this study will be shared by the lead contact upon request.
• All original code, including training scripts, pre-computed prototype embeddings, GPT-4o-generated technique descriptions, ERASER evaluation scripts, TextFooler attack scripts, and experiment configuration files, has been deposited in the CSEM_CTI GitHub repository and is publicly available as of the date of publication. Accession links are listed in the key resources table.
• Any additional information required to reanalyze the data reported in this study is available from the lead contact upon request.

## Acknowledgments

The authors extend their appreciation to the Deanship of Scientific Research at Northern Border University, Arar, KSA for funding this research work through the project number “NBU-FFR-2026-2990-07”.

## Author contributions

Conceptualization, J.P. and A.I.; methodology, J.P., S.T., and N.K.; software, J.P.; investigation, J.P., S.T., and A.A.; formal analysis, J.P. and N.K.; data curation, S.T.; resources, A.A.; writing – original draft, J.P.; writing – review and editing, S.T., A.A., N.K., and A.I.; visualization, J.P.; funding acquisition, A.A. and N.K.; supervision, A.I.; project administration, A.I. All authors read and approved the final manuscript.

## Declaration of interests

The authors declare no competing interests.

## Declaration of generative AI and AI-assisted technologies in the writing process

During the preparation of this work, the authors used GPT-4o (OpenAI) for the offline generation of technique-discriminative prototype descriptions, as described under STAR Methods. GPT-4o was not used for writing, classification, or evaluation. After using this tool, the authors reviewed and edited the content as needed and take full responsibility for the content of the publication.

## STAR★Methods

### Key resources table

**Table:** 

REAGENT or RESOURCE	SOURCE	IDENTIFIER
**Deposited data**		

SeqMask Dataset	GitHub	https://github.com/MuscleFish/SeqMask
LLM-TIKG Dataset	GitHub	https://github.com/Netsec-SJTU/LLM-TIKG-dataset
CTIBench Dataset	GitHub	https://github.com/maveryn/cti-bench

**Software and algorithms**		

GPT-4o	OpenAI	https://openai.com/index/hello-gpt-4o/
TextFooler	GitHub	https://github.com/jind11/TextFooler

### Method details

#### Problem formulation

Let X denote the space of input CTI sentences and Y the ATT&CK technique label space. CSEM-CTI learns a partition function *g*(*X*, *θ*) that decomposes the token space of an input X∈X into a minimal evidence set *X*^+^ and its noise complement *X*^−^ such that (i) *f*(*X*^−^) carries zero predictive information (necessity), (ii) *f*(*X*^+^) recovers the predictive accuracy of *f*(*X*) (sufficiency), and (iii) the resulting partition can be evaluated under the ERASER hard-erasure protocol.^22^

#### Datasets

Three publicly available CTI corpora are used (statistics in the dataset statistics table below). The SeqMask corpus^13^ (15,300 ATT&CK-labeled procedure descriptions, 184 technique classes, 70/10/20 split) is the primary benchmark; the 20 most frequent techniques account for 61% of training labels, and 47 techniques have five or fewer training instances. The LLM-TIKG corpus^17^ (38,946 labeled instances, 229 techniques, 80/20 split) provides a cross-writing-style test. The CTI-ATE task from CTIBench^18^ (approximately 600 real 2024 malware reports, 397 techniques) provides a zero-shot evaluation with no CSEM-CTI fine-tuning. All corpora are sentence-level.

**Table:** Dataset statistics used in this work

Corpus	Instances	Tactics	Techniques	Split	Role
SeqMask^13^^,^^14^	15,300	14	184	70/10/20	Primary
LLM-TIKG^17^	38,946	14	229	80/20	Cross-corpus
CTI-ATE^18^	∼600	14	397	Test only	Zero-shot

#### Contextual encoding module

SecureBERT^31^ is a 12-layer transformer pre-trained on 1.23 million cybersecurity documents (masked-language-model perplexity 6.47 on held-out CTI text, versus 28.9 for general BERT-base). The encoder produces contextualized token embeddings $H\in R^{w\times 768}$ with sequence length *w* = 128. The encoder is frozen during the warm-up phase and then jointly fine-tuned with a layer-wise learning-rate schedule:

(Equation 1) $$\eta_{\ell }=\eta_{0}\;\cdot \;\lambda^{L-\ell },\;\eta_{0}=2\times 10^{-5},\;\lambda =0.95,\;L=12.$$

#### LLM-guided prototype initialization (offline, phase 0)

For each ATT&CK technique *T*_*j*_ in the corpus label set, GPT-4o (gpt-4o-2024-11-20; temperature 0.2; top-p 1.0; max tokens 200) is prompted to generate a three-sentence description of the minimal linguistic markers signaling that technique, explicitly excluding the technique name and identifier. Each description is embedded using SecureBERT to produce $c_{j}^{\left(0\right)}$. The number of prototypes per tactic is selected by minimizing BIC:

(Equation 2) $$K_{t}^{*}=\arg\;\underset{K}{\min}BIC\left(K\right),\;t=1,\ldots ,14,$$

yielding 23 prototypes across all 14 tactics. Each description was spot-checked for length compliance, omission of identifiers, and presence of an operational indicator; the sampling temperature of 0.2 was selected from a sweep over {0.0, 0.2, 0.5, 0.8} as the value minimizing Q1 variance.

The full system prompt used for all generations is as follows.

“You are a cybersecurity threat intelligence analyst with deep expertise in the MITRE ATT&CK framework. Your task is to describe, in three sentences, the minimal linguistic evidence that appears in unstructured threat intelligence reports when a specific ATT&CK technique is present. Focus only on observable signals: action verbs, object types, and contextual phrases that an analyst would point to as evidence. Do not provide conceptual definitions, background explanations, or generic commentary. Do not mention the technique name, technique identifier, sub-technique identifier, or any ATT&CK metadata anywhere in your response. Your response must be exactly three sentences and must be written in professional analyst style.”

The user prompt is a single line in which the technique name is substituted into a placeholder: “Describe the minimal linguistic evidence pattern for the ATT&CK technique: {TECHNIQUE_NAME}”. For example, for the PowerShell sub-technique the user prompt becomes: “Describe the minimal linguistic evidence pattern for the ATT&CK technique: PowerShell (T1059.001).”

Two example generated descriptions, used directly as prototype seeds, are reproduced below to illustrate the style of the outputs. For technique T1059.001 (PowerShell), the generated description reads: “Threat reports describe adversaries invoking a scripting shell to execute encoded commands or download payloads from remote servers. The text typically references bypass flags for execution policy restrictions and base64-encoded strings passed to an interpreter. Behavioral indicators include spawning child processes from a parent interpreter or piping output to a local file for subsequent execution.”

For technique T1566.001 (Spear-phishing Attachment), the generated description reads: “Reports describe the delivery of a weaponized document or archive as an email attachment to a targeted recipient, typically framed with a plausible business context such as an invoice, resume, or policy update. The text references macro-enabled files, LNK shortcuts, or archive containers that unpack upon interaction, and notes that the message often impersonates a trusted sender. Behavioral indicators include Office processes spawning script interpreters, the extraction of embedded OLE objects, and outbound network requests triggered from within an opened document.”

#### Evidence partitioning

Token importance is computed as

(Equation 3) $$s_{i}\left(h\right)=-\|\left(h-c_{i}\right)⊙W_{i}{\|}_{2},\;s\left(h\right)=\underset{i}{\sum }a_{i}\cdot s_{i}\left(h\right),$$

and converted to a continuous differentiable mask

(Equation 4) $$s^{+}\left(h,\theta \right)=\sigma \left(2d\;\cdot \;s_{\text{norm}}\left(h,\theta \right)-d\right),\;d=4.0,$$

yielding **H**^+^ = **H** ⊙ *s*^+^(*h*, *θ*) and **H**^−^ = **H** − **H**^+^.

#### Contrastive necessity loss

The MAE necessity loss in SATG is replaced with an InfoNCE objective:

(Equation 5) $$L_{3,NCE}=-\frac{1}{n}\underset{i}{\sum }\log\frac{\exp\;\left(sim\left(f\left(H_{i}^{+}\right),y_{i}\right)/\tau \right)}{\sum_{k}\exp\;\left(sim\left(f\left(H_{k}^{-}\right),y_{k}\right)/\tau \right)},$$

with temperature *τ* = 0.07 and batch size 64 (63 in-batch negatives per positive). This loss provides a variational lower bound on the mutual information *I*(**H**^+^; *Y*) that tightens with batch size and does not equal MI for finite batches.^36^

#### Hierarchical focal classification

A tactic gate predicts the ATT&CK tactic

(Equation 6) $$g_{t}=softmax\;\left(W_{\text{tac}}\;\cdot \;h_{\text{CLS}}\left(H^{+}\right)+b_{\text{tac}}\right),$$

restricting technique prediction to approximately 16 compatible techniques. Within the restricted space, a frequency-weighted focal loss^38^^,^^39^

(Equation 7) $$L_{\text{focal}}=-\underset{j}{\sum }\alpha_{j}{\left(1-{\hat{p}}_{j}\right)}^{2}\;y_{j}\log\;{\hat{p}}_{j},\;\alpha_{j}=1/\sqrt{{freq}_{j}},$$

concentrates gradient on rare classes.

#### Total objective and training procedure

The total objective is

(Equation 8) $$L=L_{1}+L_{2}+L_{3,NCE}+\beta \;\cdot \;L_{\text{focal}},\;\beta =0.5,$$

with *L*_1_ = CE(*y*, *f*(**H**)) and *L*_2_ = ‖*f*(**H**) − *f*(**H**^+^)‖_1_ ⋅ max(0, *θ*_*l*_). Training proceeds in three phases. Phase 0 is offline prototype initialization. Phase 1 freezes SecureBERT and trains the evidence mining and classification modules with *L*_1_ + *L*_2_ for epochs 1 to 6, with curriculum *α* annealing linearly from −4.0 to 0.0. Phase 2 jointly fine-tunes all modules for epochs 7 to 20, with *α* annealing linearly from 0.0 to −2.0. The *α* endpoints were chosen by a grid search over { − 5.0, − 4.0, − 3.0} (lower bound) and { − 2.5, − 2.0, − 1.5} (upper bound), using validation technique macro-F1 as the selection criterion. Optimization uses AdamW (weight decay 0.01) with gradient clipping at max-norm 1.0 and fp16 mixed precision; early stopping uses patience 5 on validation macro-F1. Full hyperparameter settings are listed in the hyperparameter settings table below.

**Table:** CSEM-CTI hyperparameter settings

Parameter	Value	Description
Encoder	SecureBERT-base	Pre-trained on 1.23M CTI docs
Sequence length	128 tokens	Extended from 64 in^14^
Prototype count	23 (BIC per tactic)	Selected per tactic
Masking strategy	Percent-M	Retained from^14^
InfoNCE *τ*	0.07	Temperature
Focal *γ*	2.0	Focusing exponent^38^
Focal *β*	0.5	Loss weight
Learning rate (SecureBERT)	2 × 10^−5^	Layer-wise decay *λ* = 0.95
Learning rate (EMM+HCM)	5 × 10^−4^	AdamW, weight decay 0.01
Gradient clip	max-norm 1.0	–
Epochs/warm-up	20/6	–
Curriculum *α* Phase 1	−4.0 to 0.0	Linear, epochs 1–6
Curriculum *α* Phase 2	0.0 to −2.0	Linear, epochs 7–20
ERASER threshold	*s*^+^ ≥ 0.5	Post-training empirical (not tuned)
Random seeds	{42, 123, 456, 789, 1024}	Five independent runs
CPU/RAM	Core i9-14900K/128 GB	32 logical CPUs
GPU/VRAM	RTX 4080/16 GB	CUDA 12.6
Precision	fp16 mixed	–

#### ERASER protocol

The hard-erasure protocol^22^ is applied to the SeqMask test set (*n* = 3, 060): tokens in **H**^+^ are replaced with zero vectors. The evidence set is defined as all tokens with importance *s*^+^(*h*, *θ*) ≥ 0.5 after the trained Percent-M masking strategy, which corresponds on average to 32% of non-padding tokens per sentence. This 32% figure is a post-training empirical observation and was not optimized against the ERASER metrics. Comprehensiveness is Comp = Acc(*X*) − Acc(*X* \ *X*^+^) and sufficiency is Suff = Acc(*X*^+^).

#### Adversarial robustness protocol

TextFooler^41^ is configured as untargeted with maximum substitutions min(10, ⌊0.20 × *L*⌋) per sentence, counter-fitted-embedding cosine similarity threshold 0.84, and search budget 50 candidates per word. CTI-specific tokens (CVE identifiers, SHA/MD5 hashes, tool names, PowerShell and shell commands, IP addresses, file paths) are excluded from substitution via a regex-based token filter. No adversarial training or synonym augmentation was used during CSEM-CTI training.

### Quantification and statistical analysis

All results are reported as mean ± standard deviation over five independent runs with random seeds {42, 123, 456, 789, 1024}. Statistical significance for CSEM-CTI versus SATG P-M comparisons is assessed using McNemar’s paired test on prediction vectors, with Bonferroni correction across multiple comparisons at family-wise *α* = 0.01. The total number of test instances on SeqMask is *n* = 3, 060. ERASER comprehensiveness and sufficiency are reported as the mean across the SeqMask test set; the stability column in Table 4 reports the standard deviation of per-token importance scores across the five seeds. Cross-corpus evaluation uses single-pass inference on the corresponding test sets with no fine-tuning re-runs. Per-tactic gate statistics in Table 9 are computed by stratifying SeqMask test predictions by gold tactic. Definitions of significance levels, sample sizes, and dispersion measures appear in each figure and table legend.
